# In vitro antioxidant properties of the biflavonoid agathisflavone

**DOI:** 10.1186/s13065-018-0443-0

**Published:** 2018-06-29

**Authors:** Anderson Wilbur Lopes Andrade, Keylla da Conceição Machado, Katia da Conceição Machado, Daiana Dias Ribeiro Figueiredo, Jorge Mauricio David, Muhammad Torequl Islam, Shaikh Jamal Uddin, Jamil A. Shilpi, Jéssica Pereira Costa

**Affiliations:** 1 Laboratory of Research in Experimental Neurochemistry, Federal University of Piauí (UFPI), Teresina, Brazil; 2 Chemistry Institute, Federal University of Bahia (UFBA), Salvador, Brazil; 3 Department for Management of Science and Technology Development, Ton Duc Thang University, Ho Chi Minh City, Vietnam; 4 Faculty of Pharmacy, Ton Duc Thang University, Ho Chi Minh City, Vietnam; 5 Pharmacy Discipline, School of Life Sciences, Khulna University, Khulna, 9208 Bangladesh

**Keywords:** *Caesalpinia pyramidalis*, Radical scavenging, Reducing power, Lipid peroxidation

## Abstract

**Purpose:**

Free radicals are considered as the causative agents of a variety of acute and chronic pathologies. Natural antioxidants have drawn attention of the researchers in recent years for their ability to scavenge free radicals with minimal or even no side effects. This study evaluates the antioxidant capacity of agathisflavone, a naturally occurring biflavonoid by a number of in vitro methods.

**Methods:**

Agathisflavone was subjected to DPPH, ABTS, OH and NO radical scavenging assay, reducing potential and inhibition of lipid peroxidation (TBARS) test using trolox as a standard.

**Results:**

Agathisflavone showed concentration-dependent antioxidant activity against all types of free radicals used in this study. The antioxidant capacity, reducing potential and inhibition of lipid peroxidation showed by agathisflavone were comparable to that of trolox.

**Conclusion:**

Agathisflavone exhibited antioxidant capacity, which suggests considering this biflavonoid for the use in the prevention and/or treatment of diseases precipitated by oxidative stress.

## Introduction

Free radicals, both reactive oxygen and nitrogen species (ROS/RNS), produced by partial reduction of oxygen and nitrogen respectively, are inevitable processes of our body [1–3]. Some of these ROS and RNS are involved in various fundamental processes of cell survival, including mitochondrial respiration and regulation, signalization, cell proliferation and differentiation. However, any alteration in the cellular levels of the ROS/RNS can induce oxidative stress leading to cellular damages, hampering proper cellular functioning or even cell death [4, 5]. Prolonged exposure to ROS/RNS results in the damages to cell macromolecules such as carbohydrates, proteins, lipids as well as genetic materials (e.g., DNA, RNA) which eventually gives rise to the pathophysiology of a number of serious diseases including cancer, diabetes, atherosclerosis, immunosuppression, swelling, cardiovascular diseases and neurodegenerative disorders [6, 7]. Evidence suggests that certain neurological disorders such as, Alzheimer’s disease, Parkinson’s disease, schizophrenia, anxiety and depression have a direct link to long term exposure of the cells to excessive oxidative stresses [8]. In this context, naturally occurring antioxidants have been studied extensively to find their possible role in the prevention and cure for such diseases [9, 10].

Flavonoids represent an important class of natural antioxidants with significant therapeutic potential. Biflavanoids, a type of flavonoids, comprehends a large group of compounds with promising effect against oxidative damage [11].

**Fig. 1:**
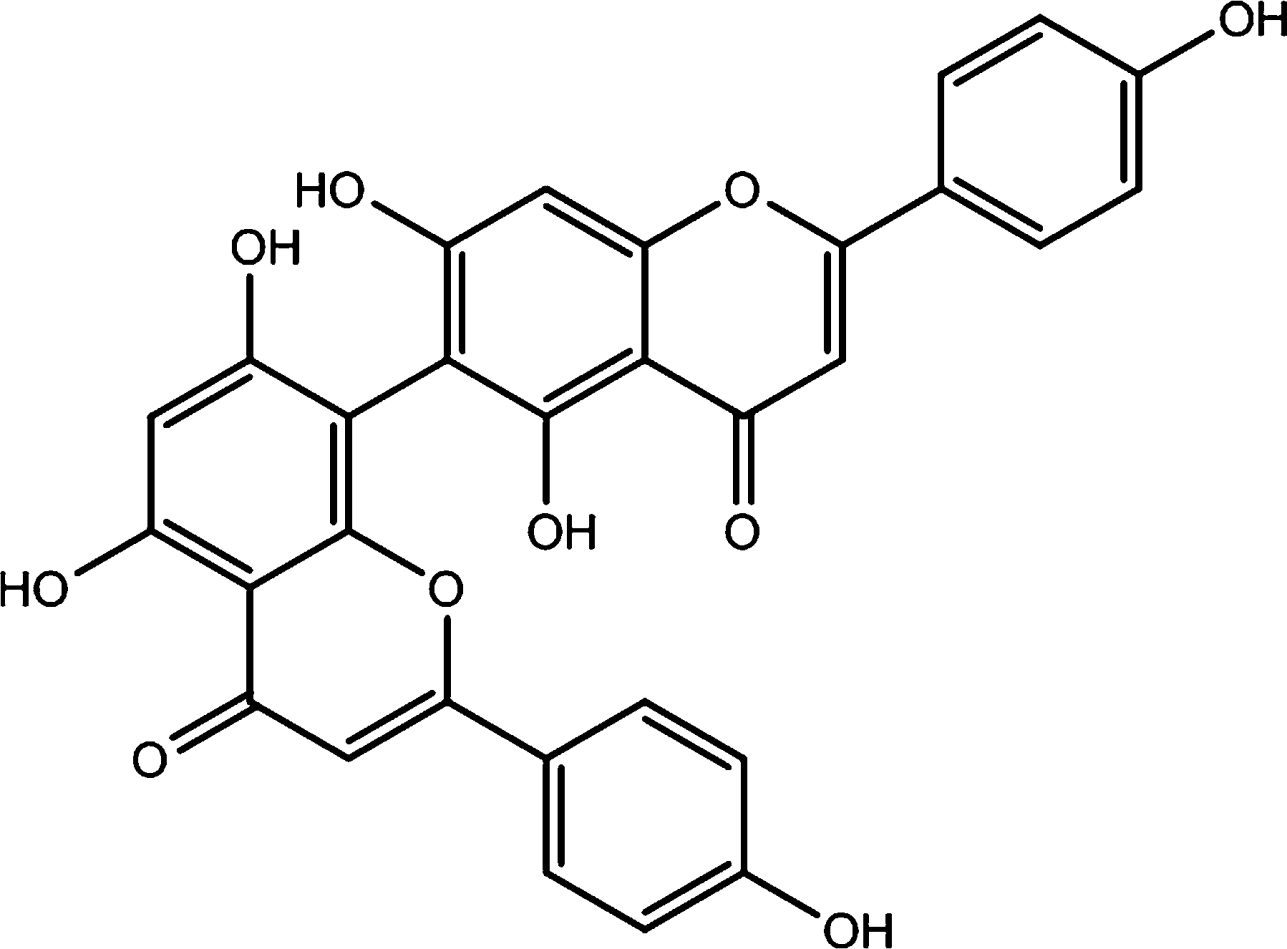
Chemical structure of agathisflavone

The biflavonoids are structurally dimer of flavonoids connected with each other by a C–C or C–O glycosidic linkage [12]. Same as those flavonoids, bioflavonoids have been reported for a number of biological activities proved through in vitro and ex vivo studies, which include anti-inflammatory [13–15], inhibition of cytochrome P_450_ enzymes [16], antiviral [17, 18], and neuroprotective activity [19, 20]. Agathisflavone, chemically known as (8-[5,7-dihydroxy-2-(4-hydroxyphenyl)-4-oxochromen-6-yl]-5,7-dihydroxy-2-(4-hydroxyphenyl)chromen-4-one) is a yellow color biflavonoid with the molar mass of 538.457 g/mol, density 1.656 g/cm^3^ and splitting coefficient 5.09 (Fig. 1) [21]. It occurs in different parts (e.g. leaf, stem, fruit, root) of many plants including *Caesalpinia pyramidalis*, *Anacardium occidentale*, *Rhus parviflora* and can be extracted by polar or medium polar solvents including methanol and ethanol [22–25]. Previous studies suggest that agathisflavone has some important biological activities, including antiviral [26], antimicrobial [27], and neuroprotective [28] activity. However, little research was done with the intent of describing the antioxidant property of this compound.

Thus the present study was designed to assess the antioxidant property of this biflavonoid by evaluating its ability to scavenge the free radicals, namely DPPH, ABTS, hydroxyl and nitric oxide (NO). It was also tested for its capacity to inhibit lipid peroxidation by TBARS method and the ability to transfer electron by ferric reducing assay.

## Materials and methods

### Chemicals

Trolox, 2,2-diphenyl-1-picrylhydrazyl (DPPH), 2,20-azinobis(3-ethylbenzthiazoline-6-sulfonic acid) (ABTS), thiobarbituric acid (TBA), trichloroacetic acid (TCA), sodium nitroprusside (SNP), 2,20-azobis-2-amidinopropane dihydrochloride (AAPH), 2-deoxyribose and potassium ferricyanide were purchased from Sigma-Aldrich Co. (St. Louis, MO, USA). All chemicals and solvents used were of analytical grade.

### Plant

Fresh leaves of *Caesalpinia pyramidalis* Tull. (Family: Fabaceae) was collected from Valente, Brazil and identified by Prof. Dr. L. P. de Queiróz (State University of Feira de Santana) and Prof. Dr. M. de F. Agra (Laboratory of Pharmaceutical Technology UFPB). A voucher specimen was deposited at the herbarium of Alexandre Leal da Costa of Biology Institute of the Federal University of Bahia (accession number 240291).

### Extraction, isolation and identification of agathisflavone

Fresh leaves of *C. pyramidalis* was extracted with methanol and chromatographed using silica gel to get pure agathiflavone. The structure was elucidated by spectroscopic data including 1D, 2D NMR and LS–MS carried out at the Department of Chemistry, Federal University of Bahia, Brazil (data not shown). Noteworthy to mention here that naturally occurring agathisflavone exhibits atropisomerism and thus was isolated as a mixture of the atropisomers.

### In vitro antioxidant activity evaluation

#### DPPH radical scavenging test

This test was done according to the method described by Machado et al. [29] with slight modifications. Briefly, a reaction mixture containing of 0.5 mL of agathisflavone (0.058, 0.116, 0.232, 0.464 and 0.928 mM) was mixed with 1.4 mL of DPPH stock solution in ethanol (100 µM). Resulting solution was mixed vigorously and kept in the dark at room temperature for 30 min. Same procedure was followed for trolox used as standard and the absorbance was measured for both agathisflavone or trolox against control at 517 nm. The percentage of inhibition was calculated by the following equation:

$$ \% \;Inhibition\;of\;DPPH = \left( {\frac{{A_{c} - A_{t} }}{{A_{c} }}} \right) \times 100 $$

where A_c_ and A_t_ is the absorbance of the control and test (agathisflavone/trolox), respectively.

#### ABTS radical scavenging test

The method described by Re et al. [30] with slight modifications was used for this test. The ABTS radical cation was initially formed by mixing 5 mL of 7 mM ABTS with 88 µL of 2.45 mM of potassium persulfate (K_2_S_2_O_8_) solution with further incubation at room temperature in the absence of light for 16 h. The resulting solution was diluted in ethanol in such a way to obtain an absorbance of 0.70 ± 0.05 at 734 nm. This test was done in the dark and at room temperature. An aliquot (0.5 mL) of agathisflavone/trolox (0.058–0.928 mM) solution was mixed with 1.96 mL of the ABTS solution and the absorbance was measured after 6 min. The results were expressed as percentage of inhibition of the ABTS in a similar fashion to that of DPPH radical scavenging assay.

#### OH radical scavenging assay

Hydroxyl radical (OH·) was generated by Fenton reaction [31] with slight modifications. Different concentrations (0.058–0.928 mM) of agathisflavone/trolox at was added to the reaction medium containing FeSO_4_ (6 mM), 2-deoxyribose (5 mM), H_2_O_2_ (100 mM) and phosphate buffer (20 mM, pH 7.4). After incubating the mixture for 30 min at ambient temperature, the reaction was terminated by the addition of phosphoric acid (4%, w/w), followed by the addition of 1% TBA (50 mM, NaOH aqueous solution). It was then heated for 15 min at 95 °C, cooled and the absorbance was measured at 734 nm in a spectrophotometer. The results were expressed as percentage of 2-deoxyribose degradation.

$$ \% \;2{\text{-}}Dexyribose\; degradation\; = \left( {\frac{{A_{c} - A_{t} }}{{A_{c} }}} \right) \times 100 $$

where A_c_ and A_t_ is the absorbance of the control and test (agathisflavone/trolox), respectively.

#### Reducing potential assay

The method described by Singhal et al. [32] with slight modifications was used to determine the reducing capacity of agathisflavone. Briefly, 1 mL of agathisflavone/trolox (0.058–0.928 mM) was added with 1 mL of 1% potassium ferricyanide and 0.5 mL of sodium phosphate buffer (0.2 M, pH 6.6). The reaction mixture was incubated at 50 °C for 20 min, followed by the addition of 0.5 mL of 10% TCA, 0.5 mL of distilled water and 0.25 mL of 0.1% ferric chloride. The absorbance of the reaction mixture was measured at 700 nm in a spectrophotometer against a blank solution. The EC_50_ of the sample and standard required for 50% reduction potential was determined.

#### NO scavenging test

Nitric oxide was produced from the spontaneous decomposition of sodium nitroprusside in 20 mM phosphate buffer (pH 7.4). Once generated, nitric oxide interacts with oxygen to produce nitrite ions, which was measured by the Griess reaction [33]. The reaction mixture containing 1 mL of SNP in phosphate buffer (20 mM) and 0.5 mL of agathisflavone/trolox (0.058–0.928 mM) was incubated at 37 °C for 1 h. An aliquot (0.5 mL) of the reaction mixture was taken and homogenized with 0.5 mL of Griess reagent. Absorbance of chromophore was measured at 540 nm in a spectrophotometer and the results were expressed as percentage inhibition of nitrite ions.

#### Inhibition of lipid peroxidation (TBARS test)

The TBARS (thiobarbituric acid reactive substances) method described by Guimarães et al. [34] was used evaluate the ability of agathisflavone to inhibit lipid peroxidation. Briefly, an aliquot of (0.5 mL) of egg yolk homogenate (5% w/v in 50 mM phosphate buffer, pH 7.4) was mixed with 0.5 mL of agathisflavone/trolox (0.058–0.928 mM). The lipid peroxidation was induced by the addition of 0.5 mL of AAPH (200 mM) for 60 min at 37 °C. Subsequently, 1 mL of TCA (10%) and 1 mL of TBA (0.67%) was added and heated at 97 °C for 15 min. After 15 min, the reaction mixture was centrifuged and the absorbance of the supernatant was measured at 532 nm. The extent of lipid peroxidation was estimated by TBARS levels formed and the results were expressed as percentage of inhibition of lipid peroxidation:

$$ \% \;Inhibition\;of\;lipid\;peroxidation = \left( {\frac{{A_{c} - A_{t} }}{{A_{c} }}} \right) \times 100 $$

where A_c_ and A_t_ is the absorbance of the control and test (agathisflavone/trolox), respectively.

### Statistical analysis

All the experiments were done in triplicate and the results are expressed as mean ± standard error of mean (SEM). Statistical analysis was performed using the program GraphPad Prism^®^ 6.02 (San Diego, CA, USA), one-way ANOVA, multiple comparisons following by *t*-Student–Newman–Keuls post hoc test. The results were considered statistically significant at p < 0.05.

## Results

### DPPH radical scavenging

**Fig. 2:**
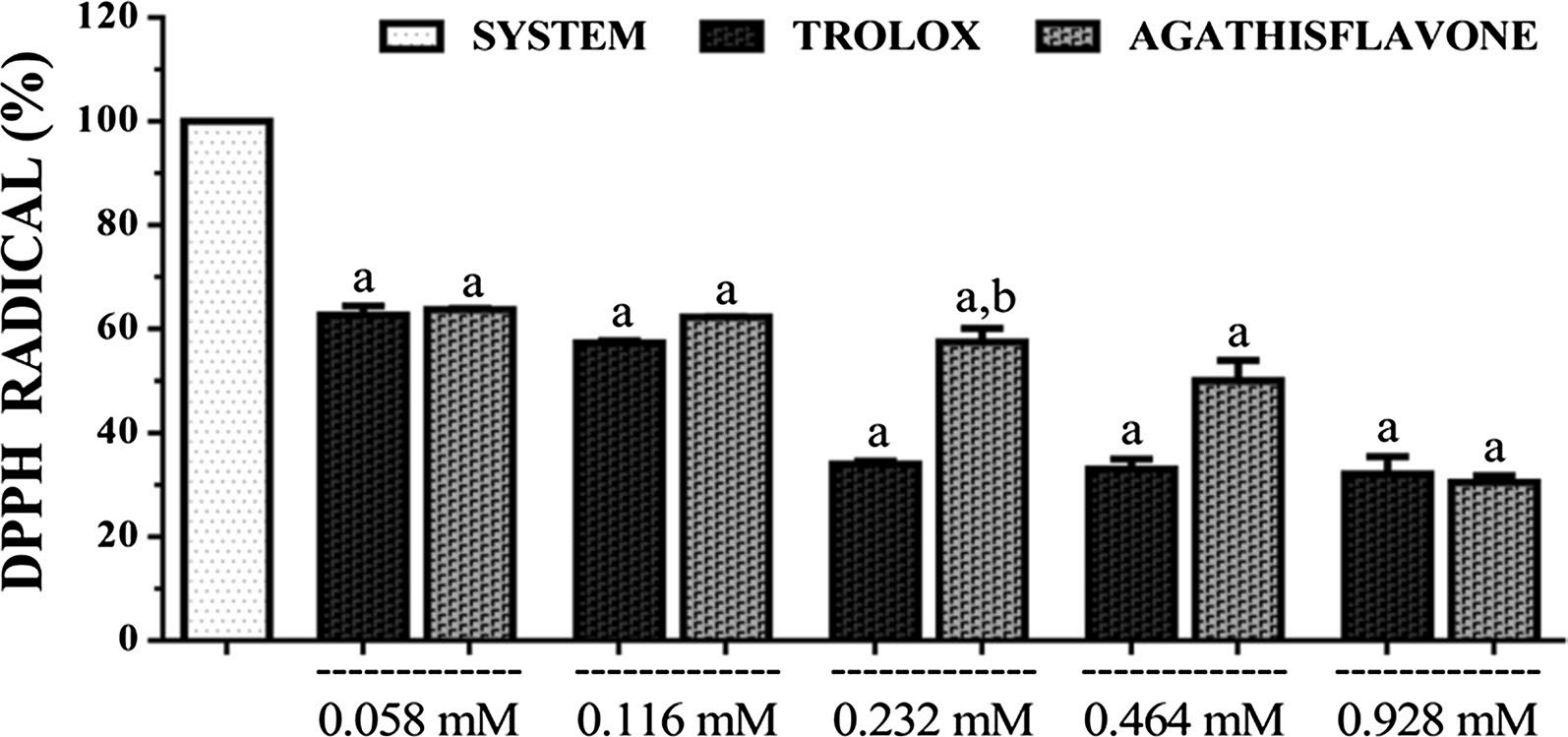
DPPH· scavenging capacity of agathisflavone and trolox. [Values are mean ± SEM (n = 3) ^a^p < 0.05 when compared to the system (100% of DPPH·), ^b^p < 0.05 when compared to the trolox (standard) (ANOVA and *t*-Student–Neuman–Keuls as a post hoc test)]

Agathisflavone was found to scavenge DPPH radical in a concentration-dependent manner with the highest inhibition observed for the highest concentration of agathisflavone tested (0.928 mM). The radical scavenging capacity of agathisflavone was almost similar to that of the trolox at all concentrations (except at 0.232 and 0.464 mM). The EC_50_s calculated for agathisflavone and trolox were 0.474 mM (0.399–0.564 mM) and 0.149 mM (0.129–0.173 mM), respectively at 95% confidence interval (Fig. 2).

### ABTS scavenging assay

**Fig. 3:**
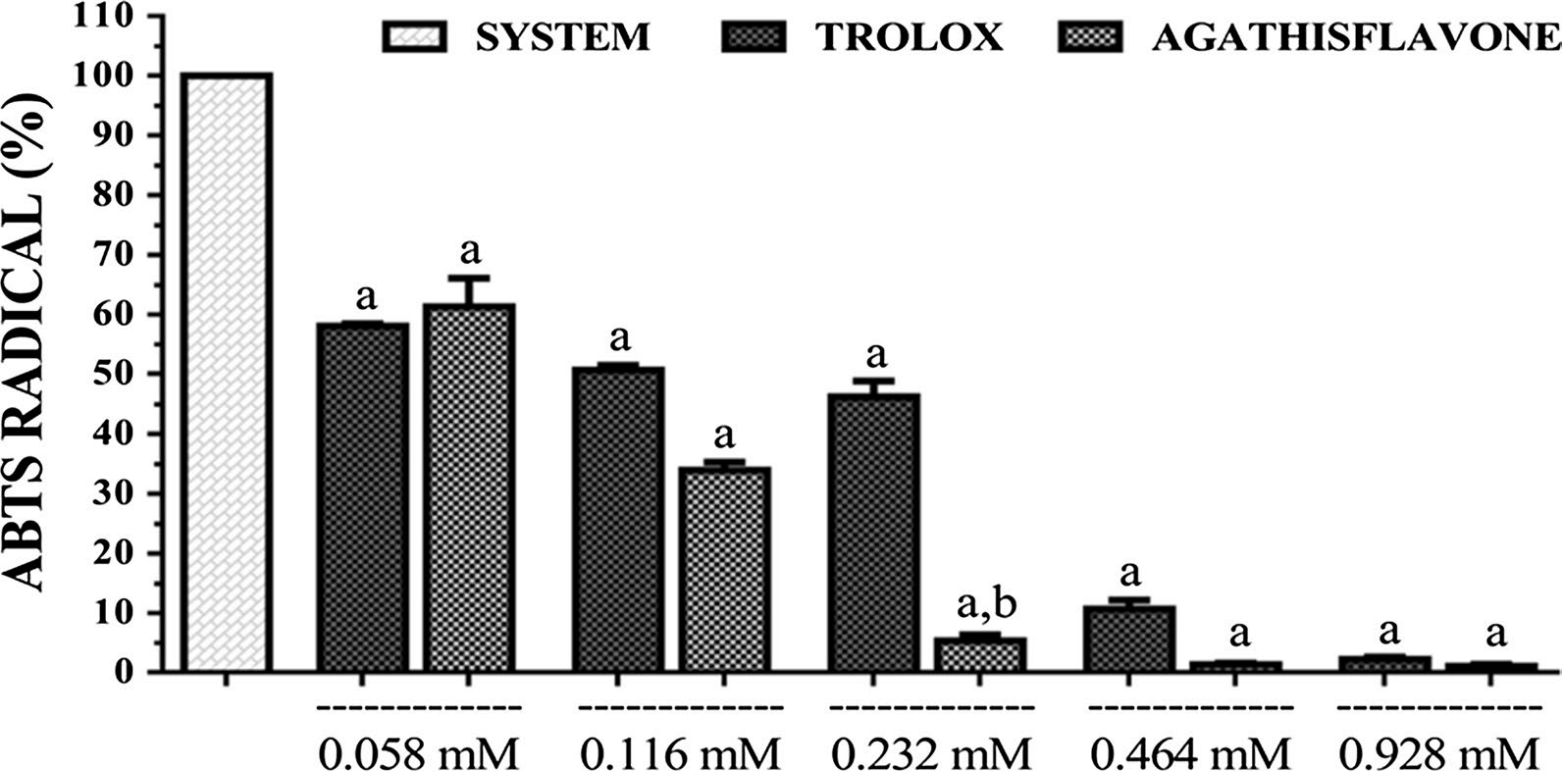
ABTS radical scavenging capacity of agathisflavone and trolox. [Values are mean ± SEM (*n* = 3); ^a^p < 0.05 when compared to the system (100% of ABTS), ^b^p < 0.05 when compared to trolox (ANOVA and *t*-Student–Neuman–Keuls as a post hoc test)]

Concentration dependent ABTS radical scavenging activity was observed for both agathisflavone and trolox with the highest inhibition observed for the highest concentration tested (Fig. 3). EC_50_s calculated for agathisflavone and trolox were 0.179 mM (0.137–0.234 mM) and 0.311 mM (0.283–0.341 0.474 mM), respectively with 95% confidence interval.

### OH radical scavenging assay

**Fig. 4:**
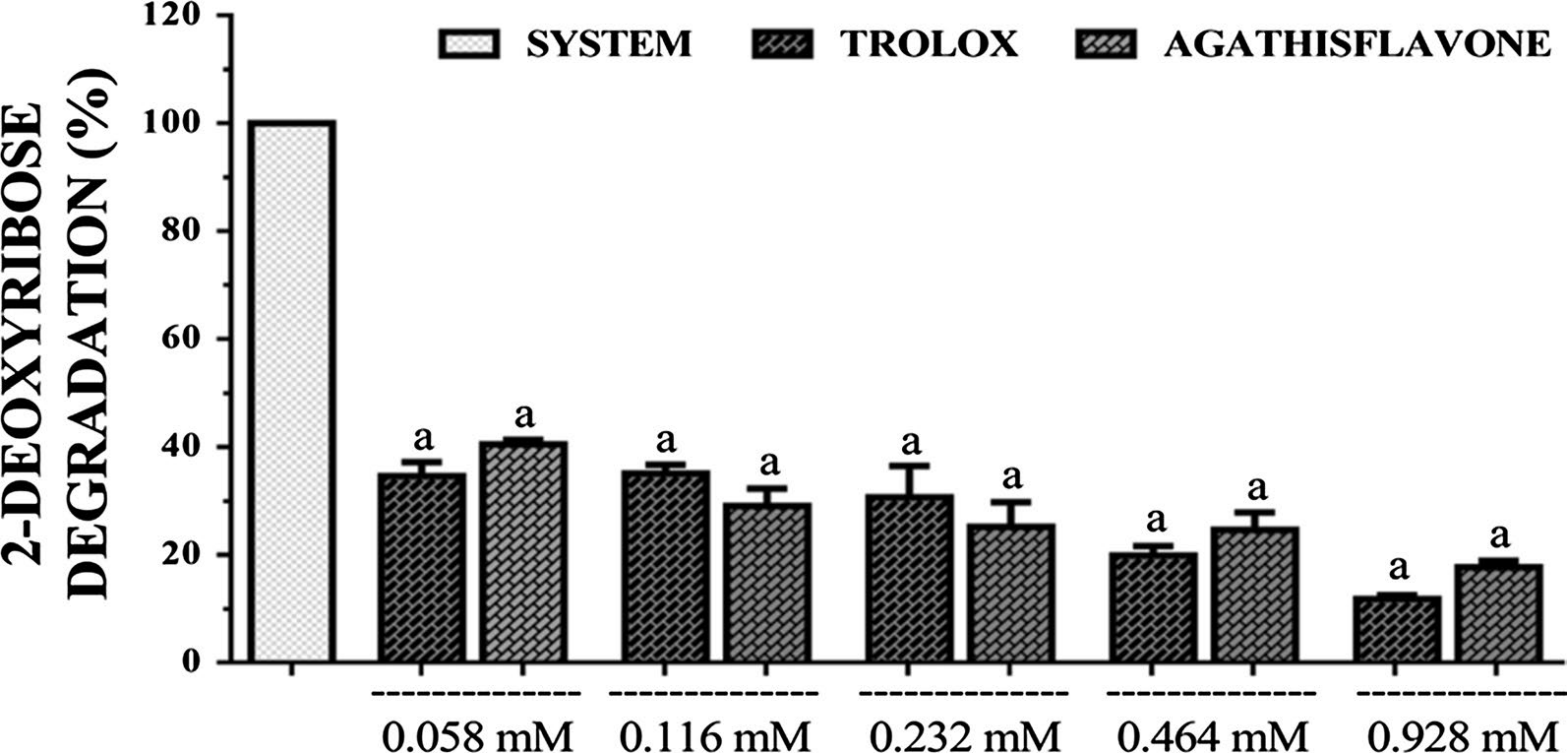
OH· scavenging capacity of agathisflavone and trolox. [Values are mean ± SEM (*n* = 3) ^a^p < 0.05 when compared to the system (100% of OH·), ^b^p < 0.05 when compared to the trolox (standard) (ANOVA and *t*-Student–Neuman–Keuls as a post hoc test)]

In this study, agathisflavone showed a concentration-dependent OH radical scavenging capacity. The activity was slightly lower than that of trolox only at 0.116 and 0.232 mM, but higher at other concentrations with statistical significance (p < 0.05) (Fig. 4). EC_50_s calculated for agathisflavone and standard were 0.163 mM (0.101–0.263 mM) and 0.372 mM (0.280–0.496 mM), respectively with 95% confidence interval.

### Reducing potential

**Fig. 5:**
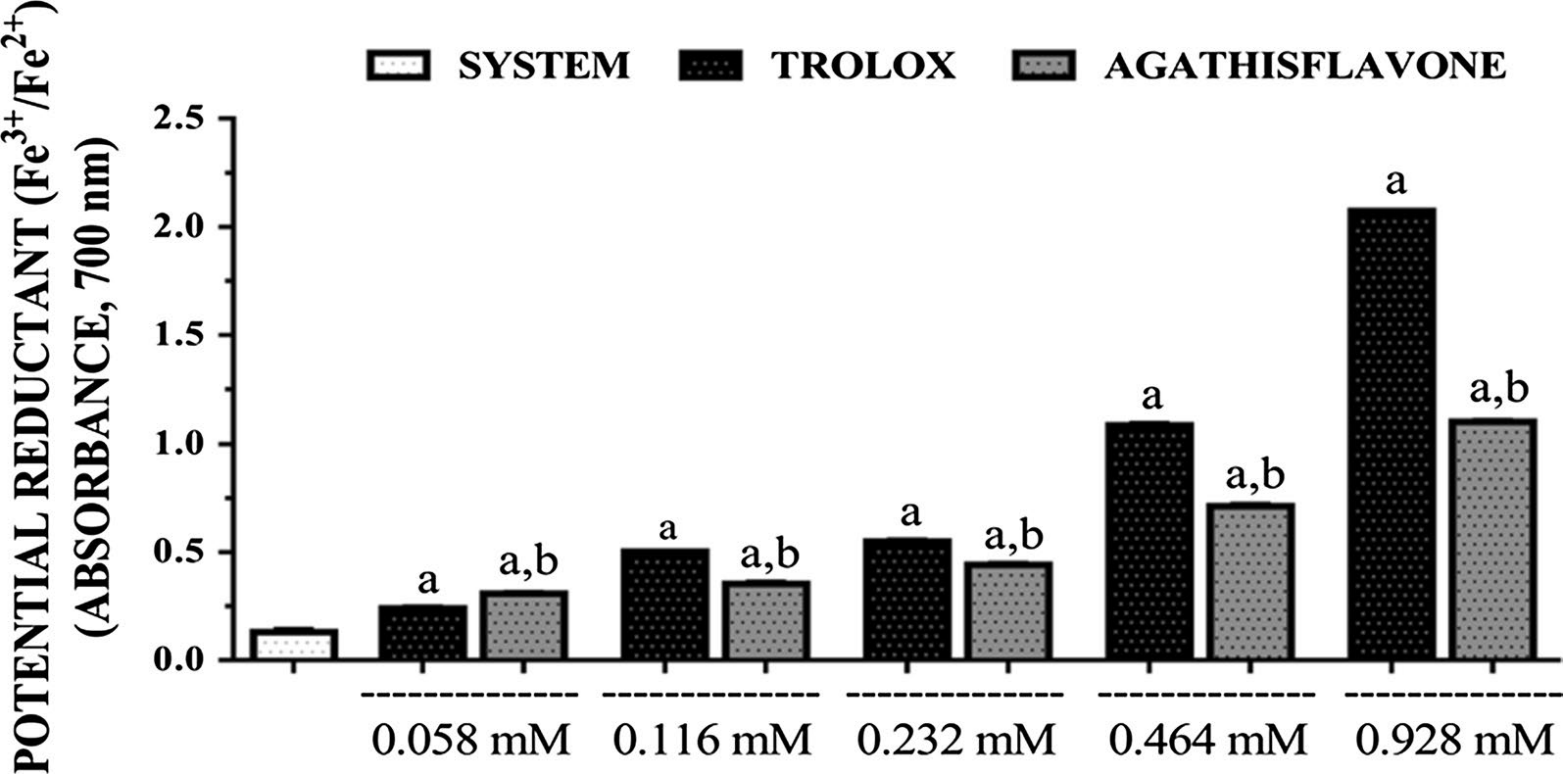
Reducing potential (Fe^3+^/Fe^2+^) of agathisflavone and trolox. [Values are mean ± SEM (*n* = 3) ^a^p < 0.05 when compared to the system (without agathisflavone/trolox), ^b^p < 0.05 when compared to the trolox (standard) (ANOVA and *t*-Student–Neuman–Keuls as a post hoc test)]

Both agathisflavone and trolox reduced ferric ion to ferrous in a concentration dependent manner (Fig. 5). The activity was slightly higher for trolox than that of agathisflavone. The EC_50_s calculated for the sample and standard were 0.163 mM (0.101–0.263 mM) and 0.372 mM (0.280–0.496 mM), respectively with 95% confidence interval.

### NO scavenging assay

**Fig. 6:**
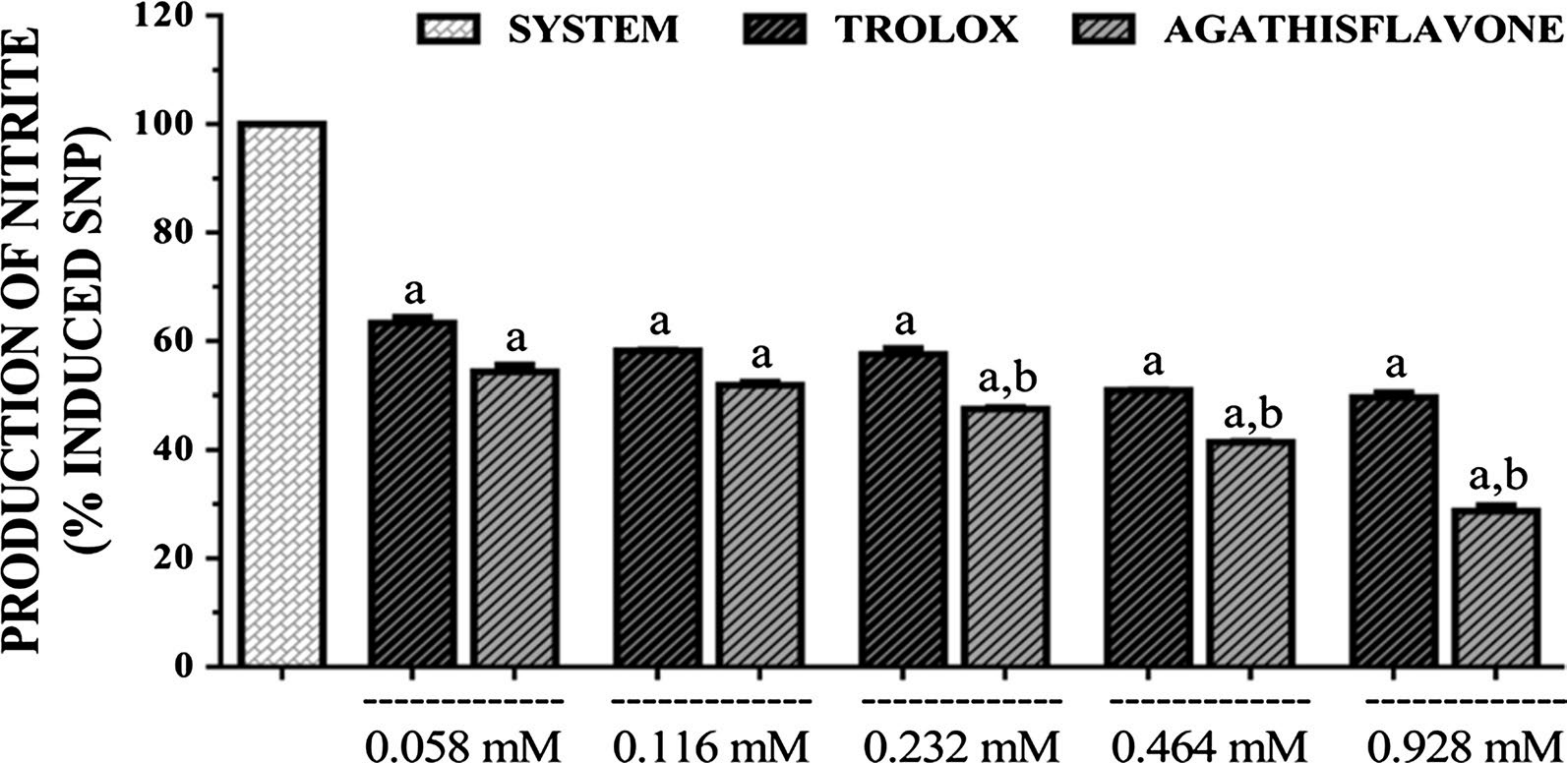
NO radical scavenging capacity of agathisflavone and trolox. [Values are mean ± SEM (*n* = 3) ^a^p < 0.05 when compared to the system (100% of nitrite ions), ^b^p < 0.05 when compared to the trolox (standard) (ANOVA and *t*-Student–Neuman–Keuls as a post hoc test)]

Agathisflavone exhibited prominent NO scavenging activity which was evident from the reduced production of nitrite ion (Fig. 6). Concentration-dependant antioxidant NO scavenging capacity was observed for both agathisflavone and standard and the activity was slightly higher for agathisflavone that that of trolox. EC_50_s calculated for the sample and standard were 0.209 mM (0.162–0.2682 mM) and 0.456 mM (0.415–0.493 mM), respectively with 95% confidence interval.

### TBARS test

**Fig. 7:**
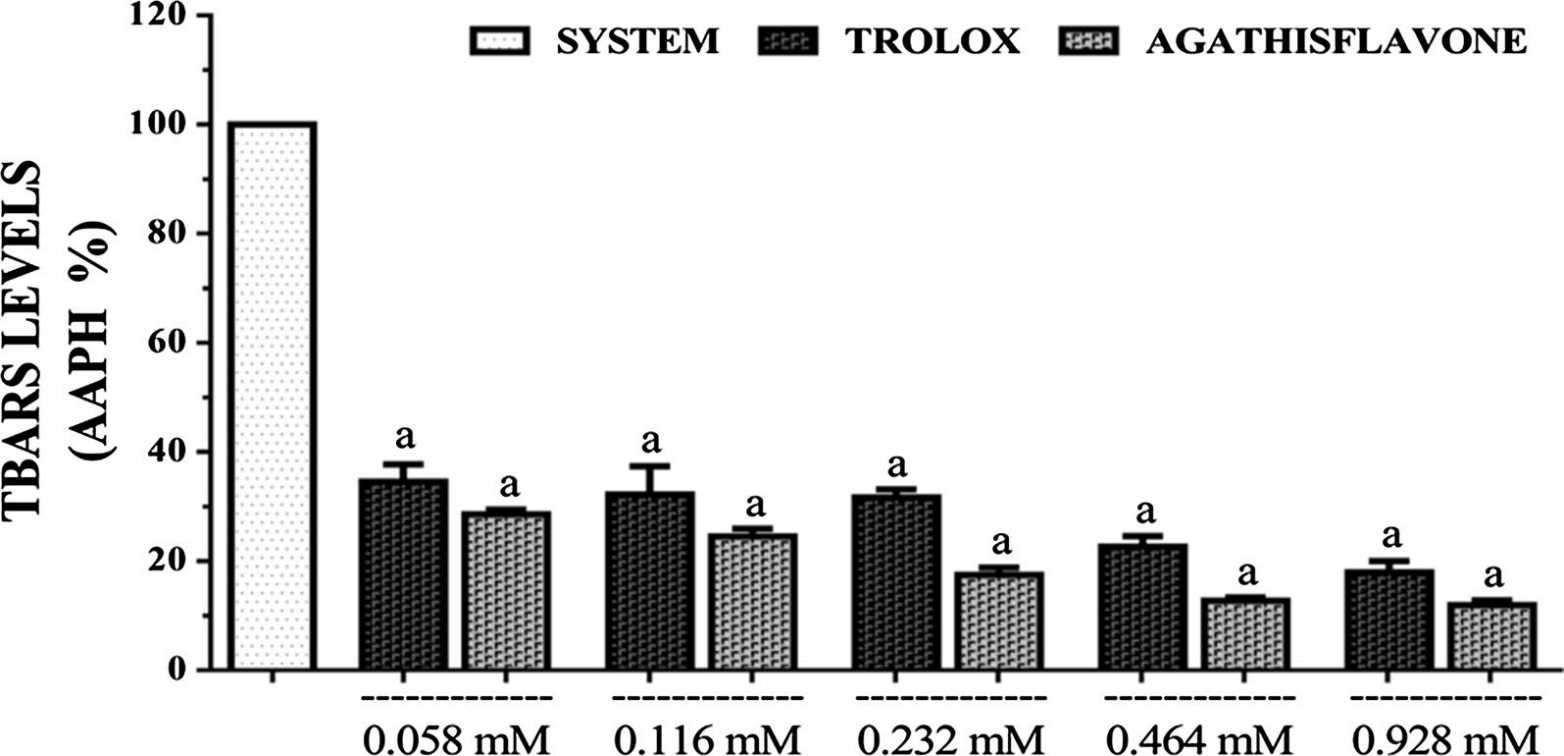
TBARS inhibitory capacity of agathisflavone and trolox. [Values are mean ± SEM (*n* = 3) ^a^p < 0.05 when compared to the system (100% of TBARS levels), ^b^p < 0.05 when compared to trolox (standard) (ANOVA and *t*-Student–Neuman–Keuls as a post hoc test)]

In this test, agathisflavone was found to be a better inhibitor of lipid peroxidation that that of trolox at all the concentrations tested (Fig. 7). Both agathisflavone and trolox showed concentration-dependent inhibition of lipid peroxidation evident from the reduced levels of EC_50_s calculated for agathisflavone and standard were 0.179 mM (0.154–0.208 mM) and 0.352 mM (0.233–0.530 mM), respectively with 95% confidence interval.

## Discussion

**Fig. 8:**
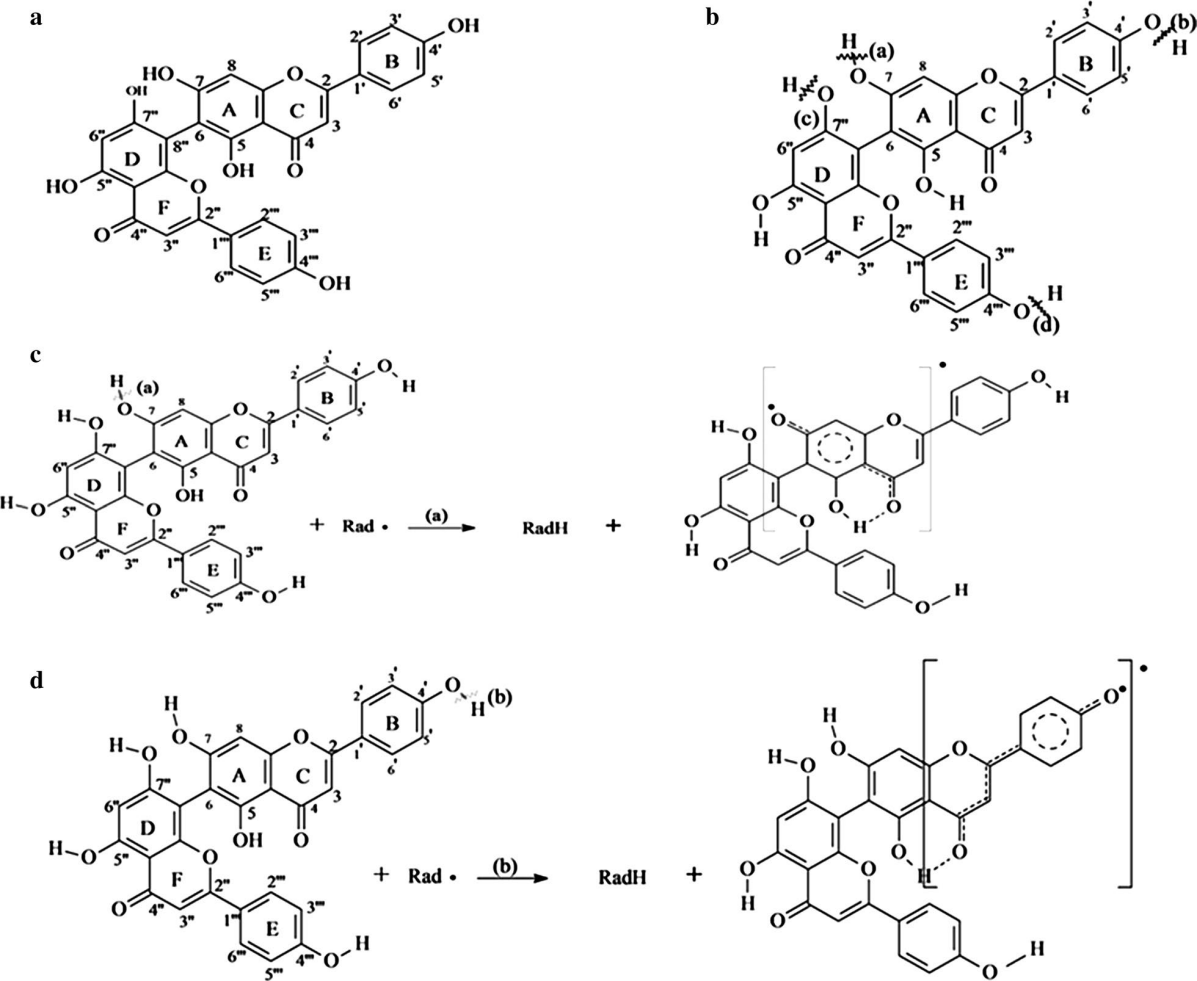
Proposed mechanism of antioxidant activity of the agathisflavone by donating of H· and electrons. **a** Chemical structure of agathisflavone (OH groups at various positions). **b** Possible C-terminals from where proton (H^+^) can be donated. **c** and **d** Proton (H^+^) donation and neutralization of free radicals, resonance structures of agathisflavone

Present investigation suggests that agathisflavone possesses prominent antioxidant capacity as observed from several in vitro antioxidant test systems. There are four hydroxyl groups in the structure of agathisflavone (Fig. 8a), i.e., 4′–OH, 7–OH, 7″–OH and 4‴–OH, (Fig. 8b) that can donate H· (radical hydrogen) to reduce free radicals. Although the rest two hydroxyls (5″–OH and 5–OH) radicals cannot donate radical hydrogen, but they do participate in the process by forming resonance structures (Fig. 8c, d).

Antioxidant activity of several flavonoids with mechanism similar to that of agathisflavone have been studied using methods as DPPH and/or ABTS, OH and NO scavenging assay, as well as their ability to inhibit lipid peroxidation by TBARS assay and reducing ability [35, 36].

When compared, agathisflavone was found to be a better scavenger of ABTS than that of DPPH. Although both ABTS and DPPH are free radicals, but the differ in the way that DPPH is a stable free radical itself but ABTS is formed instantly in the reaction solution. Thus both of the methods are used for antioxidant activity study but a difference in the EC_50_ of a compound can occur due to the difference in the mechanism of action of neutralizing free radicals. In relating the results of DPPH· (EC_50_ = 0.895 mM) and ABTS^·+^ (EC_50_ = 0.123 mM) tests, it is possible to say that agathisflavone showed a better scavenging capacity of ABTS^·+^. This may be due to effects of donation of electrons, leading to the reduction of ABTS^·+^ formation. On the other hand, transference of hydrogen atoms may occur to form of a DPPH stable molecule by the action of this biflavonoid [37, 38]. Our findings are similar to the results suggested by Ye et al. [39], who were isolated a biflavonoid from the methanolic extract of the *Camellia oleifera* Abel shells and with a chemical structure that of the agathisflavone.

Agathiflavone isolated from the leaf extract of *Anacardium occidentale* was subjected to DPPH radical scavenging assay by Ajileye et al. [25]. The EC_50_ value calculated for agathisflavone was 366.37 μg/mL, which is equivalent to 0.679 mM, slightly lower that the value observed in the present work (0.474 mM). This slight change may happen due to the variation of the DPPH· concentration in the reaction mixture.

The compounds that have large quantities of ·OH (free) in their chemical structures have higher reducing potential [11]. In a previous study the antioxidant activity of the natural biflavonoid (morelloflavone-4000-*O*-b-d-glicosil, fukugiside e morelloflavone) isolated from the ethyl acetate extract of the dried fruits of *Garcinia brasiliensis* was seen with the potential reduction capacity [9]. Like the evaluated compound in this study, the biflavonoids isolated by Gontijo et al. [9] showed reducing capacities in a concentration-dependent manner.

The ·OH oxidizing radical and its presence in the reaction medium promotes degradation of 2-deoxyribose. In our study, agathisflavone at all concentration tested may react with ·OH, thus the inhibition of the degradation of the monosaccharide utilized [3, 40]. Based on the comparison between the EC_50_ values of the agathisflavone and the antioxidant standard (trolox) in inhibiting the 2-deoxyribose degradation, this biflavonoid can be considered as a potent scavenger of ·OH. This may be an indication of protecting important biomolecules, such as proteins, lipids and genetic materials (e.g.—DNA, RNA) [41].

Agathisflavone also significantly (p < 0.05) inhibited the levels of NO. An excessive generation of NO is related to a number of pathological conditions, including intracellular oxidative damages and cell death [13, 41]. Thus, the inhibitory effects of this damaging radicals may inhibit or protect cells and cellular organelles from the damaging effects of NO. Furthermore, peroxyl radicals generated by AAPH are evident to cause lipid peroxidation [42]. In our study, we found that the agathisflavone significantly inhibited TBARS production in comparison to the NC and trolox, suggesting a prominent protective capacity of the lipid molecules from oxidative damage.

The biflavonoids procyanidin, fukugetin, amentoflavone and podocarpusflavone isolated from the ethyl acetate extract of the leaves of *Garcinia brasiliensis* have been found to exhibit antioxidant capacity at 10 µM (equivalent to 0.01 mM) with an average inhibition by 28, 42, 37 and 30%, respectively, those values are smaller than the activity observed in the quercetin group (100 µM; 47%) [5]. In this study, we found an average inhibition for the agathisflavone at 0.928 mM by 88%. It seems, the biflavonoid agathisflavone may be a potent antioxidant.

## Conclusion

The antioxidant capacity investigated of the agathisflavone applying in vitro test systems allows to conclude that the this biflavonoid delay or prevent significantly the lipid peroxidation and the other referred molecules induced by free radicals, since, the compound showed an antioxidant capacity in DPPH·, ABTS^·+^, OH·, NO and reduction potential tests. The oxidative damage is linked to inducing damages in the brain and other organs, leading to varieties of health effects in human and other animals. The agathisflavone may be a new hope in the context of drug discovery and development, especially with the importance of the prevention and treatment of diseases related to oxidative stress.
